# Young people’s decisions about biologic therapies: who influences them and how?

**DOI:** 10.1093/rheumatology/keu523

**Published:** 2015-02-05

**Authors:** Ruth I. Hart, Helen E. Foster, Janet E. McDonagh, Ben Thompson, Lesley Kay, Andrea Myers, Tim Rapley

**Affiliations:** ^1^Institute of Health and Society, Newcastle University, ^2^Institute of Cellular Medicine, Newcastle University, Newcastle, ^3^School of Immunity and Infection, University of Birmingham, Birmingham, ^4^Musculoskeletal Services, Newcastle Hospitals NHS Foundation Trust, Newcastle upon Tyne, and ^5^Rheumatology, Northumbria Healthcare NHS Foundation Trust, North Shields, UK

**Keywords:** young people, inflammatory arthritis, decision making, biologic therapies, trusted others, interdependence, qualitative research

## Abstract

**Objectives.** Young people with inflammatory arthritis can have severe disease warranting biologic therapy. They face complex treatment decisions, with profound consequences. This study aimed to explore the influence of individuals outside the care team (trusted others) on the treatment decisions made by young people, in particular their decisions about biologic therapies.

**Methods.** Young people (16–25 years of age) with inflammatory arthritis and experience of treatment decision making were recruited from three NHS Hospital Trusts. Twenty-five were interviewed, plus 11 trusted others identified by young people as being involved in their decision making, as well as 6 health professionals. The data were analysed using coding, memoing and mapping techniques and the findings were tested through a series of focus groups.

**Results.** Young people initially emphasized their decisional autonomy, typically describing people other than health professionals as limited in influence. However, discussions revealed the involvement—in deliberation and enactment—of a range of other people. This cast of trusted others was small and largely consistent; mothers played a particularly prominent role, providing cognitive, practical and emotional support. Members of the wider cast of trusted others were involved in more limited but still significant ways.

**Conclusion.** Young people claim autonomy but other people enable this. The network of relationships in which they are embedded is distinctive and evolving. Mothers play a supporting role well into early adulthood; in contrast, partners are involved in far more limited ways. As such, the applicability of adult models of decision making is unclear. This must be taken into account if the support provided by professionals is to be optimally tailored to young people’s needs.

**Rheumatology key messages**

- Young people claim autonomy in treatment decision making, but other people, especially mothers, enable this.
- Other people’s influence on young people’s treatment decisions may be significant without being obvious.
- In promoting independent decision making, care teams should take relationships with trusted others into account.

## Introduction

Recent years have seen important changes in the clinical management of inflammatory arthritis, in particular the widespread use of biologic therapies in both paediatric and adult services. Young people with aggressive disease are more commonly being offered such treatments, and at an earlier stage in the disease course [1]. The evidence is clear that short-term benefits can be considerable, and include reductions in joint pain and damage, plus improved mobility. However, there are also short-term risks (e.g. increased vulnerability to infection), and the long-term consequences of these treatments (e.g. impact on fertility, risk of malignancy) remain uncertain [2]. This is of particular concern for those who begin taking them early in life. Young people offered biologics are therefore confronted with a decision that may have profound consequences, at a point when their disease is at its worst and their wider lives are characterized by change and uncertainty.

Health care professionals play an important role as providers of information and advice for patients generally [3] and for young people specifically [4, 5]. However, treatment decisions have also been shown to be influenced by interactions with people outside the health care team [6, 7]. In considering lay influence, the research literature focuses substantially on the significant other, with this term typically connoting a long-term partner or spouse [8, 9]. Yet in the UK the trends are clear: people are committing to a partner much later than in the past [10, 11]. If a growing proportion of young adults do not have a significant other, focussing exclusively on this relationship as a source of influence or support for decision making is problematic. We therefore looked more broadly at the who, how and why (or why not) of lay involvement in young people’s treatment decisions. We refer to this broader group of people as trusted others.

## Methods

We report here on one component of a wider study of young people’s decision making regarding biologics. That study employed a range of qualitative methods: interviews (with young people, trusted others and health professionals), recording of patient/professional interactions and focus groups. The analysis reported here draws principally on data from interviews, but is informed by learning from other study strands and was validated in the concluding focus groups. The study conformed to National Institute for Health Research requirements and had Research Ethics Committee approval from the Proportionate Review Sub-committee, National Research Ethics Service Committee Yorkshire & Humber—Leeds East (ref. 12/YH/0122). All participants gave consent verbally and in writing.

### Setting

Potential interviewees (and participants in other research strands) were identified and recruited via three NHS Hospital Trusts, two in the North East of England and one in the West Midlands. These trusts operated one or more of the following rheumatology services: adult clinics, young adult clinics run by adult and/or paediatric rheumatologists with interests in adolescence and adolescent clinics run by paediatric rheumatologists.

### Sample

Our approach to sampling was purposive, seeking to encompass variation in demographic characteristics, diagnosis and treatment history (see Table 1) and to explore emerging conceptual issues. Requests were made to direct care colleagues to identify and approach young people with specific characteristics. Young people (*n* = 25) were between 16 and 25 years of age at the first interview and had a diagnosis of inflammatory arthritis (either JIA, AS, PsA or RA). At first contact they either had not yet been offered a biologic (*n* = 5), had recently been offered a biologic (*n* = 5) or already had some experience with one or more biologics (*n* = 15). Where young people’s treatment status changed, i.e. as they started taking a first or subsequent biologic, attempts were made to re-interview them.

**Table 1:** Characteristics of young people interviewed

Characteristic	Value
Diagnosis, *n*	
JIA	15
AS	7
PsA	2
RA	1
Gender, *n*	
Female	15
Male	10
Age, mean (range), years	20 (16–25)
Disease duration, mean (range), years	9 (<1–>20)
Rheumatology service accessed, *n*	
Adult clinic	10
Young adult clinic	8
Adolescent clinic	7

Trusted others (*n* = 11) were identified by participating young people and approached through them. Most agreed to participate and this subsample of interviewees included eight mothers, one father, one grandmother and one partner. Trusted others who declined to participate included a close friend and a partner. Health professional interviewees (*n* = 6) were identified by the core research team and chosen to include key roles within the multidisciplinary teams at the participating trusts and their service providers.

### Data collection

R.I.H. interviewed 25 young people, 5 on more than one occasion, plus 11 trusted others and 6 health professionals. None of the interviewees had encountered R.I.H. prior to the start of the project. In five cases, young people and trusted others were interviewed together; in all other instances interviewees were spoken to individually. Interviews were semi-structured, lasted 40–120 minutes and were predominantly conducted face to face at a location of the participant’s choice. Interview schedules were initially informed by the team’s experience and a review of the literature. These were adjusted to take account of individual circumstances (e.g. young people’s treatment status) and refined following each round of analysis. All interviews were recorded, transcribed verbatim and anonymized. Field notes were written after each contact and provided an additional resource for analysis.

### Data analysis

The study data comprises 52 transcripts (of which 44 relate to interviews). These were closely and systematically examined by R.I.H. using open and focused coding, mapping and memoing techniques to identify, classify, label and relate themes, phenomena and ideas [12, 13]. Data segments (selected transcripts or data pertaining to a particular theme) were similarly analysed by T.R. Analyses were compared, shared and developed further with other researchers in fortnightly data clinics, biannual team and steering group meetings and a concluding series of focus groups. These four focus groups comprised young people (*n* = 7, *n* = 3), trusted others (*n* = 4) and health professionals (*n* = 8). They were a vehicle for establishing face validity, providing a forum in which research participants and their peers could comment on the intelligibility, credibility and significance of the findings.

## Results

### My decision … but: stories of enabled autonomy

In the following sections we report on the involvement of others in young people’s treatment decision making and look in detail at four important roles these trusted others play. Overall the message is one of qualified autonomy, encapsulated by the recurrent expression ‘my decision … but’. This echoes research into young people’s experiences of treatment decision making in other clinical areas [4, 14, 15]. However, while the findings of those studies suggest constraint, the stories emerging from our data were largely about enablement.

**Fig. 1:**
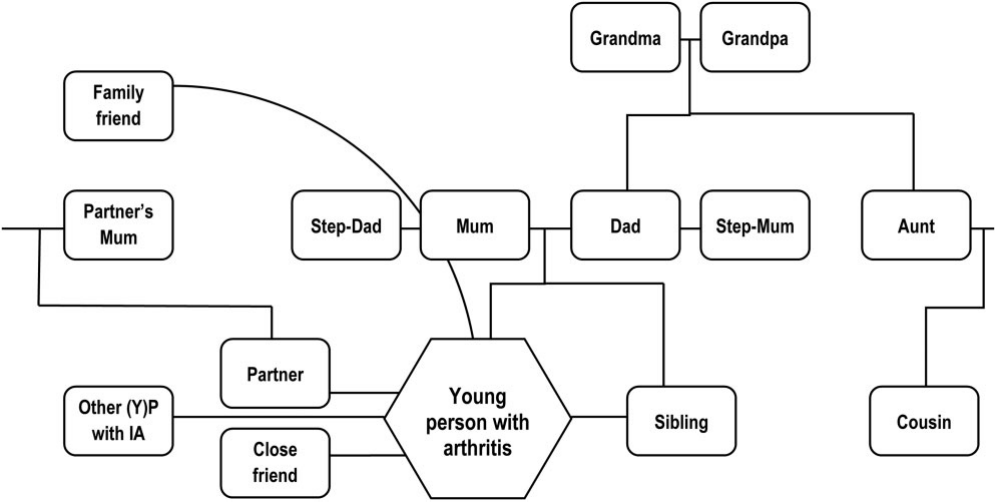
Trusted others featured in our data set

In general, young people in our study began by emphasizing their autonomy, typically describing people other than health professionals as limited in influence. However, in subsequent discussion they revealed the involvement of a number of other people in the making and making possible of treatment decisions. A mapping exercise (Fig. 1) showed this cast of individuals with influence to be relatively small and largely consistent. All relationships had foundations in the real world and the group was dominated by close family. Mothers played a particularly prominent role in the accounts, as detailed in the following subsection.

### Trusted (m)others: I’ve always had me mam there

Mothers featured prominently in stories of making and enacting decisions in around three-quarters of cases. The majority of these young people (17/20) were living under the same roof as their mother at the time of the research. In two further instances, where the young person’s mother was not able to play such a significant role, someone else (a father and a grandmother) had stepped in. The small minority of young people who did not acknowledge the role of a mother, or stand-in, had distinctive characteristics, typically having adult-onset conditions that had been diagnosed after leaving home and/or moving in with their partner. In essence, these were young people who were organizing their lives like adult patients.

**Fig. 2:**
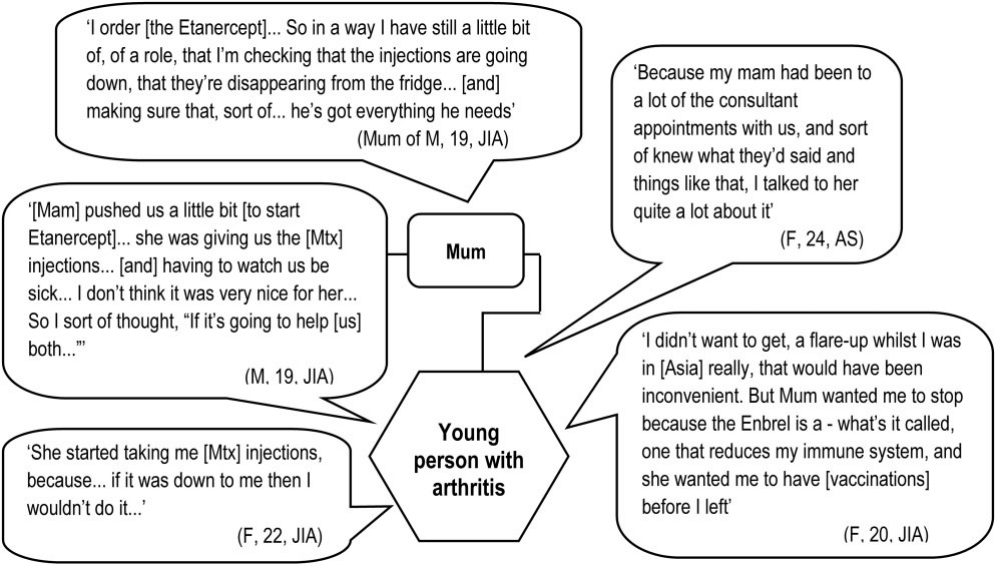
Mothers’ involvement in and influence on treatment decisions

Mothers are distinctive for the centrality of their role and for the variety of ways in which they are involved in decision making. It is common for them to be implicated in both deliberation and enactment, and their involvement spans practical, cognitive and emotional realms (see Fig. 2).

Examples of practical support include facilitating access to services and enabling (sometimes enforcing) the following through of decisions made in clinic. Young people and trusted others explained how mothers were involved in some or all of the following tasks: making appointments; taking the young person to the hospital; ordering, receiving and storing medications and preparing and in some instances administering injections. It was exceedingly rare for anyone else to be involved in the more intimate of these tasks (e.g. administering injections). Young people understood that treatment regimes impacted on their mothers as well as themselves; a change in treatment was therefore seen by some young people as having the potential to help them both.

Mothers were also portrayed as supporting and contributing to the cognitive aspects of decision making. This included prompting or asking questions, doing research, providing information and checking understanding. In many instances they accompanied the young person to consultations—as such they provided a backup or aide-mémoire and were uniquely positioned to discuss the pros and cons of the option(s) presented to the young person. They also acted as a conduit, relaying information to other members of the family on the young person’s behalf.

Mothers additionally offered emotional support to confront an important decision at a difficult time, essentially being there for the young person and providing reassurance, comfort and encouragement. However, young people’s choices regarding where to turn for emotional support were complex. They sought at times to protect their parents (who often felt responsible for decisions made, delays in diagnosis and sometimes the condition itself), looking elsewhere to meet their emotional needs.

### Wider family: in-house experts

Members of the wider (non-nuclear) family appear to have defined but decisive roles when, in addition to ties of blood or marriage, they can claim relevant professional expertise. For example, young people talked of step-parents, an aunt and a cousin who were nurses, or allied health professionals. Young people often viewed this in-house expertise as a valuable resource. These extended family members typically had limited practical involvement, but were providers of information and advice about both the young person’s condition and potential treatment options. As such, they were in a position to substantially and directly influence treatment decisions.

A young person (male, 25 years old, diagnosis of AS) first interviewed in the early stages of the project provides a good example of this. This young man had recently transferred into the Trust, though he had had the condition since his late teens. He had met his consultant just once at the time of the first interview and was taking NSAIDs. Biologics had not been discussed. However, in the interview it emerged that he already knew about them and hoped to discuss these treatments with his consultant at his next appointment. Further questioning revealed that he had learnt about this group of drugs from his cousin, the young man explaining: ‘My cousin, she’s a nurse … she’d actually written down some names of drugs to suggest … So she’s had a bit of an influence too’. A few months after that interview he saw his consultant again and subsequently began taking adalimumab. A second interview was arranged in order to explore the circumstances of the treatment offer and decision. It transpired that (like several other interviewees with the same diagnosis) he had been given a choice of biologic. The young man explained his decision as follows: adalimumab was ‘the one I was recommended from my cousin … so I just plumped for that’.

### Empathic friends: *s*omeone I can talk to

**Fig. 3:**
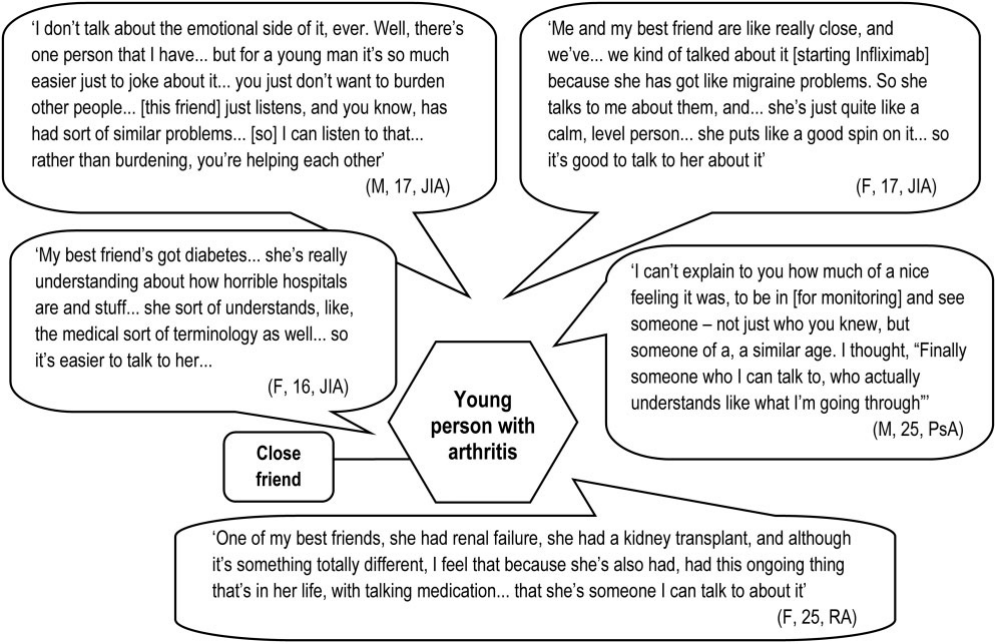
The importance of friends with similar problems

Offering a quite different—but still highly valued—kind of expertise are friends (or family members) with personal experience of ill health. These people influence orientation to new treatment options and provide support to manage the emotions associated with starting new regimes with uncertain outcomes. About a third of the young people in our sample identified one or more friends of a similar age (occasionally their boy-/girlfriend) who had their own direct experience of ill health and with whom they felt able to share quite intimate details of their illness experiences and treatment dilemmas (see Fig. 3). These friends had a variety of conditions, including kidney failure, diabetes, arthritis and migraines. They demonstrated three important qualities that set them apart from interviewees’ other peers. First, these friends had a more grounded understanding of their experience and capacity for empathy. Secondly they had knowledge of the organization of health services and medicine. Thirdly, their own difficulties provided opportunities for reciprocity. This sense of being able to help, rather than burdening each other, was clearly important to the young people in our study; this accords with the emphasis of other authors on reciprocity as the key component of friendship [16].

In contrast, young people appeared hesitant, and highly selective, regarding discussing either their treatment or condition with their wider/healthy peers. Some admitted this quite frankly. Others had their claims of being open about their condition challenged by a trusted other, subsequently qualifying their account. When probed, concern was expressed about how peers would respond. Some young people already had negative experiences of disclosure; others anticipated these. It was important to young people to be seen as normal, not as different in any significant way from their peers. However, they also talked of struggling to convey information about their condition and other people’s struggles to understand. This inability to comprehend the nature of their condition made the idea of involving healthy peers in deliberations about treatment and care look quite pointless.

### Supportive partners: *s*omeone to put the foot up your backside

Almost half of the young people interviewed mentioned a boy-/girlfriend, fiancé(e) or spouse, with six saying they were living together. However, partners rarely featured prominently in young people’s accounts of treatment decision making. No examples were found of partners providing practical support (e.g. with receipt, storage, preparation or administration of medications); where this was needed, young people turned to their mothers. Cognitive involvement also appeared modest, with young people using their partner, at most, as a sounding board. Where partners’ involvement was consistently reported was as providers of encouragement, motivation and discipline. A trusted other, whose son had lived away from home for some time, commented that people tend to get lazy with their health and the attention of someone who cares is of real value. Regarding his own son he said, ‘His partner now is, you know, nagging him more than I did—and he takes it better!’

Although young people typically said their partner knew about their condition, what that meant in practice seemed to vary considerably. It was rare for them to take their partner to hospital—just one of our interviewees reported that her boyfriend routinely attended consultations. Another said they would welcome their partner along, and some suggested they might involve their partner further if and when they considered starting a family. Most, however, expressed a clear preference to see the consultant alone, allowing them to manage the information their partner received and maintain control over any decisions made.

A few young people said they had been encouraged by health professionals to share information with their partner. They had found this advice valuable, if hard to follow. Several admitted to playing down their difficulties, saying they did not want to be fussed over or to let their partner down. So, on balance, partners appear relatively poorly informed. Fluctuations in a young person’s health, treatment regimen and responses might be observed, but are far from fully understood.

## Discussion

Young people with inflammatory arthritis who are confronted with decisions about biologic therapies vary along multiple dimensions. In addition to demographic variation, significant differences are evident in their disease trajectories and treatment histories. Despite these differences, however, we see commonalities of experience that draw them together as a group and set them apart from the more typical (older) rheumatology patient.

It is clear that while young people claim decisional autonomy, and a small minority are justified in doing so, most exhibit a relational autonomy. Their autonomy is enabled by others who shape and support the making and enactment of decisions. This is true for adults too—the literature suggests that autonomy is enabled across the life course—but different people are involved, in different ways [6, 7, 17, 18], and critically, attitudes towards their involvement are different.

Our study found that mothers often remain involved in a wide range of ways well into early adulthood, in particular—but not exclusively—where their child is diagnosed while a child. Young people in whose stories mothers (or a stand-in from within the close family) do not play a prominent role were a small and distinctive minority in our study. All had adult-onset conditions, but in addition had been diagnosed after leaving home and/or starting to cohabit with a partner. These were young people who were organizing their wider lives in an adult way.

Partners replaced parents as the first port of call in a yet smaller minority of our cases (a situation noted in other recent studies [19]). The role they take on is typically much narrower than that of mothers, and careful management of information, or partial disclosure, appears the norm. In line with previous research on disclosure (of genetic risk) in dating [20], our data suggest that sharing information with partners is seen as risk-laden and difficult. As previously pointed out elsewhere [21], insufficiency of information can cause relationship tensions and lead to misguided support for patients. Help to think through whether, when and what information to share with their partners (and indeed healthy peers) might benefit some young people both emotionally and, ultimately, clinically.

While relationships with healthy people are important [22], friendships with other people with chronic illnesses were highly valued by the young people in our study. On occasion their experiential knowledge directly informed treatment decision making. These relationships also appear to have wider and potentially lasting benefits [23, 24]. Friendship choices are rarely the preserve of individuals alone [25], hence the position taken here by care teams (and others) is important. The case for recognizing and the potential for facilitating the development of friendships with other young people with inflammatory arthritis, or chronic illnesses more widely, is worth exploring further.

The involvement of others is normal, not dysfunctional, and for patients in other age groups is largely accepted, if not entirely approved. Young people need staunch allies, and for many of the young people in our study (as suggested elsewhere [26]) their mother continues to be the best candidate. However, unlike older patients, young people are encouraged, if not required, to demonstrate independence in various ways [19]. While recognizing the importance of work to empower young patients, we believe considerable care needs to be taken to promote independence without forcing supportive relationships underground. We acknowledge that paediatric teams are increasingly working towards interdependence, where young people take responsibility for themselves but parents continue to function as consultants [27].

Our data also offer a reminder that young people cannot rely equally on their parents for support and guidance. They may be disadvantaged by family structure [28] or by resources [29]. Simmons *et al.* [15] draw our attention to young people within the care system who may have autonomy forced upon them. These, and other young people whose families are struggling or fractured, may benefit from additional professional attention and support.

Critically, interactions outside the clinical consultation matter, but while the patterns highlighted here provide a prompt to question received wisdom and taken-for-granted practices, they do not tell us who influences treatment decisions, and how, in any particular case. Hence exploring home and peer relationships using screening tools such as the Home, Education/Employment, Activities, Drugs, Sexuality, Suicide assessment tool [30] should be routine practice for all young people.

Being clear who is involved, and how, is important for several different reasons. First, it is the only way to make sure everyone involved has appropriate information—something that Elwyn *et al.* [31] have argued is a fundamental component of effective shared decision making. Secondly, such clarity equips the health care team to foresee challenges and pre-empt potential problems (e.g. on the young person moving away from home). Finally, it will help professionals identify the need for and opportunities to build independence at a pace appropriate to an individual’s needs. Fundamentally the distinctive and evolving network of relationships in which young people are embedded must be revealed and taken into account if the support provided to them by professionals is to be most effectively tailored to their needs.
